# Disentangling Li Diffusion Characteristics in Amorphous Nickel Oxide

**DOI:** 10.3390/nano16100600

**Published:** 2026-05-13

**Authors:** Chao Tang, Changlong Cai, Huachen Liu

**Affiliations:** 1 School of Optoelectronic Engineering, Xi’an Technological University, Xi’an 710021, China; tangchao_28@live.com (C.T.);; 2 School of Armament Science and Technology, Xi’an Technological University, Xi’an 710021, China

**Keywords:** nickel oxide, density functional theory, diffusion, AIMD, molecular dynamics, SGCPMD

## Abstract

The advancement of electrochromic devices, including smart windows, is important for improving energy efficiency in modern society. Nickel oxide thin films are key functional materials in this technology and have attracted significant attention due to their electrochemical activity and optical properties. However, existing theoretical studies have primarily focused on crystalline NiO, while systematic understanding of Li^+^ diffusion mechanisms in amorphous NiO remains limited. In this work, first-principles calculations combined with second-generation Car–Parrinello molecular dynamics simulations and the melt-quenching method are employed to construct amorphous NiO models with varying oxygen content, enabling investigation of oxygen-dependent Li^+^ diffusion behavior. The results show that the Li^+^ diffusion coefficient increases with increasing oxygen content, accompanied by a reduction in diffusion barriers. Analysis of local structural environments further reveals that Li coordination with under-coordinated Ni–O polyhedra plays a key role in facilitating ion migration, providing atomistic insight into the observed diffusion trends. This study establishes a structure–diffusion relationship in amorphous NiO and provides atomistic understanding of how oxygen stoichiometry modulates Li^+^ transport behavior in electrochromic materials.

## 1. Introduction

Nickel oxide (NiO) thin films are key functional materials in electrochromic devices due to their favorable optical and electrochemical properties [1]. These features make NiO widely studied in electrochromic applications, where its reversible redox activity enables optical modulation [2]. In electrochromic systems, the coloration and bleaching processes are governed by reversible Li^+^ insertion and extraction, where ion transport kinetics directly determine switching speed and optical modulation efficiency. Therefore, Li^+^ diffusion at the atomic scale plays a central role in governing electrochromic performance. Recent advancements in the study of crystalline NiO have laid a solid foundation for understanding its material properties. Experimentally, researchers have fine-tuned the oxygen content, reaction conditions, and oxidants to synthesize NiO with varying stoichiometries, ${\mathrm{NiO}}_{x}$ [3,4]. Density functional theory (DFT) has been widely used to investigate the fundamental characteristics of crystalline NiO. For instance, defect states such as oxygen vacancies have been identified as important in modifying its electronic and transport properties [5,6]. DFT+U studies have further revealed the evolution of electronic structure during lithium intercalation and its impact on optical properties [7], while other works have explored its mechanical and structural behavior [8]. Despite these advances, the amorphous form of NiO exhibits distinct local structural disorder and coordination variability that remain insufficiently understood in terms of ion transport behavior. In crystalline NiO, lithium-ion diffusion has primarily been studied in the context of defect engineering and structural optimization [9]. However, the mechanisms governing lithium-ion diffusion in amorphous NiO, particularly the role of local coordination environments and oxygen content, have not been systematically investigated. To the best of our knowledge, a systematic atomic-scale understanding of Li^+^ diffusion in amorphous NiO with varying oxygen stoichiometry remains lacking.

This work addresses this gap by employing first-principles calculations and second-generation Car–Parrinello molecular dynamics to investigate Li^+^ diffusion in amorphous NiO, revealing how oxygen content modulates ion transport through local structural environments.

## 2. Materials and Methods

To investigate the influence of oxygen content on the structure and Li^+^ diffusion in amorphous NiO (α${\mathrm{-NiO}}_{x}$, x=1,1.14,1.33), amorphous models were constructed using a melt-quenching approach. In amorphous systems, different melt-quenching realizations may lead to variations in local atomic configurations. In this work, a single equilibrated amorphous configuration is used for each composition due to the high computational cost of ab initio molecular dynamics. The reliability of the generated structures was evaluated using radial distribution functions (RDFs), coordination statistics, and time-origin averaged mean square displacement (MSD), which confirm the convergence of short-range order and the statistical stability of the trajectories. The melt-quench procedure involves heating the system to 4000 K for 10 ps, followed by cooling to 300 K over 20 ps and subsequent equilibration for 8 ps under the NPT ensemble at zero pressure. All melt-quenching simulations were performed using the second-generation Car–Parrinello molecular dynamics (SGCPMD) method [10].

Initial structures were constructed from cubic NiO (space group F$\bar{m}$3m; ICSD 646099 [11]) using a 3×3×2 supercell. Ni-deficient models were generated by randomly removing Ni atoms, resulting in α${\mathrm{-Ni}}_{72}O_{72}$, α${\mathrm{-Ni}}_{63}O_{72}$, and α${\mathrm{-Ni}}_{54}O_{72}$, corresponding to α-NiO, α${\mathrm{-NiO}}_{1.14}$, and α${\mathrm{-NiO}}_{1.33}$, with 144, 135, and 126 atoms, respectively. The system sizes are consistent with previous ab initio studies of amorphous Li-ion conductors (typically 100–150 atoms), indicating that they are sufficient to describe local structural environments relevant to ion transport [12,13]. All calculations were performed using CP2K 2023.2 [14] with the PBE functional and DZVP-MOLOPT-SR-GTH pseudopotentials [15]. The plane-wave cutoff and relative cutoff were set to 500 Ry and 60 Ry, respectively, and only the Γ point was used. The density matrix convergence threshold was set to $1\times 10^{-5}$.

For AIMD simulations, the system’s dynamics were described using Langevin dynamics with the ASPC scheme [16], combined with the melt-quench framework [17]. To ensure structural reliability, radial distribution functions (RDF) and coordination numbers were analyzed at 300 K over 1000-step trajectories. To investigate Li^+^ diffusion, 16 Li atoms were randomly inserted into the α${\mathrm{-NiO}}_{x}$ structures, followed by full relaxation using the CSVR thermostat [18]. The use of multiple Li atoms improves statistical sampling of Li^+^ hopping events within the limited AIMD timescale, while all systems maintain comparable Li number densities, ensuring that diffusion trends are not affected by Li concentration differences. Since Li^+^ diffusion is slow at room temperature, simulations were performed at 900 K, 1100 K, 1400 K, and 1700 K to accelerate diffusion sampling. Kinetic Monte Carlo (KMC) methods are not adopted in this work because amorphous ${\mathrm{NiO}}_{x}$ lacks a periodic lattice and well-defined discrete diffusion sites required for constructing transition rate catalogs. In contrast, AIMD naturally captures Li^+^ migration in a continuously disordered energy landscape without prior assumptions on hopping pathways, making it more suitable for describing diffusion in amorphous systems. At 1700 K, structural statistics were obtained from 7500 configurations extracted from the AIMD trajectories, ensuring sufficient sampling of local coordination environments.

## 3. Result and Discussion

### 3.1. Structure Parameters

The SGCPMD-based melt-quenching method has been employed to construct amorphous NiO structures to examine the influence of oxygen content on α${\mathrm{-NiO}}_{x}$. The structures are subsequently equilibrated at zero pressure using the NPT ensemble with the CSVR thermostat. Table 1 shows the structural parameters of the equilibrated configurations. The RDF and related structural statistics were obtained by averaging over 1000 equilibrium frames to ensure statistical convergence. The results indicate that as the oxygen content increases in the amorphous NiO, its volume continuously shrinks. Compared to the original supercell, the volume of the amorphous NiO shows both contraction and expansion, depending on the oxygen content. Specifically, the volumes of α-NiO and α${\mathrm{-NiO}}_{1.14}$ increase by 12.67% and 5.17%, respectively, whereas the volume of α${\mathrm{-NiO}}_{1.33}$ decreases by 7.58%. In terms of density, a similar trend is observed. The original crystalline NiO has a density of 6.74 g/cm^3^, whereas the amorphous phases show reduced densities: 5.99 g/cm^3^ for α-NiO, 5.78 g/cm^3^ for α${\mathrm{-NiO}}_{1.14}$, and 5.46 g/cm^3^ for α${\mathrm{-NiO}}_{1.33}$. This trend suggests that increasing oxygen content in the amorphous structure leads to a lower atomic packing density, consistent with the observed volume changes.

**Figure 1:**
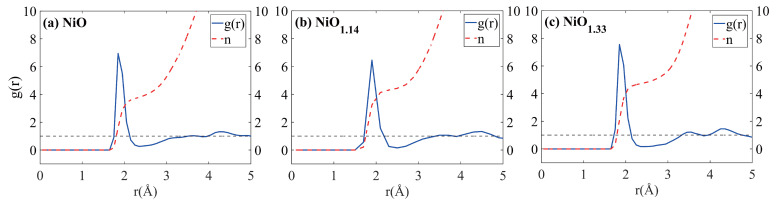
The RDF and coordination number for the Ni-O pair of (**a**) α-NiO, (**b**) α${\mathrm{-NiO}}_{1.14}$, and (**c**) α${\mathrm{-NiO}}_{1.33}$ at 300 K. The dashed line marks g(r) = 1, the average density.

Figure 1 displays the results of the calculated and analyzed Ni-O radial distribution function (RDF) for the ${\mathrm{NiO}}_{x}$ models. As observed in Figure 1a–c, the absence of long-range periodic peaks beyond the first coordination shell. suggesting that the obtained structures are amorphous ${\mathrm{NiO}}_{x}$ structures. This further supports the conclusion that the ${\mathrm{NiO}}_{x}$ structures obtained using the SGCPMD method are indeed amorphous. Moreover, for NiO, ${\mathrm{NiO}}_{1.14}$, and ${\mathrm{NiO}}_{1.33}$, the Ni-O bond length is consistently 1.875 Å. This can be attributed to the partially filled Ni 3d orbitals, which participate in hybridization with the oxygen p orbitals. The redistribution of electrons through this d–p orbital hybridization strengthens the covalent bonding between Ni and O atoms, thereby stabilizing the Ni–O bond length.

As indicated in Table 2, the Ni-O bond length in α${\mathrm{-NiO}}_{x}$ is approximately 1.875 Å, which is in good agreement with reported experimental values (1.93 Å [19] and 1.75 Å [20] depending on local coordination environments). With the increase in oxygen content, the Ni coordination numbers in NiO, ${\mathrm{NiO}}_{1.14}$, and ${\mathrm{NiO}}_{1.33}$ gradually increase, reaching 3.792, 4.362, and 4.716, respectively. This indicates that the variation in oxygen content within the NiO structure significantly influence the local structure. This suggests enhanced Ni–O hybridization, which may influence the local electronic environment and diffusion energetics.

To further assess the structural representativeness of the constructed amorphous models, additional analysis of structural descriptors was performed. The RDFs exhibit broadened first-neighbor peaks and the absence of long-range periodicity beyond the first coordination shell, together with broadened peak features, which are characteristic features of amorphous materials. At the same time, the position of the first Ni–O peak remains close to experimentally reported bond lengths, indicating that the local bonding environment is physically reasonable despite the structural disorder. Furthermore, the coordination number distributions reveal a diversity of local environments, reflecting the inherent heterogeneity of amorphous NiO. Importantly, consistent trends in coordination number and density are observed across different oxygen compositions, suggesting that the structural characteristics are not dependent on a specific configuration. These results demonstrate that the constructed models capture the essential short-range order and structural disorder of amorphous NiO and can be considered representative for investigating Li^+^ diffusion behavior.

### 3.2. Diffusivity of Lithium Ions in Amorphous α${\mathrm{-NiO}}_{x}$

Through AIMD simulations, the α${\mathrm{-NiO}}_{x}$ structure was successfully obtained, and its reliability was validated using the radial distribution function (RDF). The Li^+^ diffusion coefficient ($D_{Li^{+}}$) of α${\mathrm{-NiO}}_{x}$ plays a crucial role in anodic coloration material performance. To investigate the effect of oxygen content on Li^+^ diffusion, AIMD simulations were performed using the α${\mathrm{-NiO}}_{x}$ structure. From the Li^+^ trajectories obtained from molecular dynamics simulations, the mean square displacement (MSD) was calculated as:

(1) $$MSD\left(t\right)=\frac{1}{N}\langle \sum_{i=1}^{N}{\left|r_{i}\left(t+t_{0}\right)-r_{i}\left(t_{0}\right)\right|}^{2}\rangle ,$$

where $t_{0}$ represents multiple time origins along the trajectory.

**Figure 2:**
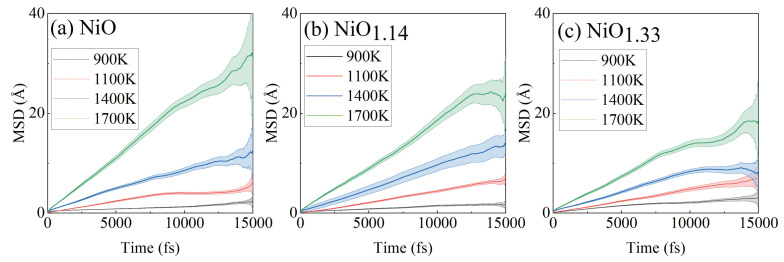
(**a**) α-NiO, (**b**) α${\mathrm{-NiO}}_{1.14}$, (**c**) α${\mathrm{-NiO}}_{1.33}$ MSD of Li^+^ at 900 K, 1100 K, 1400 K, 1700 K.

To improve statistical reliability, the MSD was averaged over multiple time origins (sliding window averaging), which enhances convergence in finite-time AIMD simulations. Figure 2a–c show the relationship between the mean square displacement (MSD) and time for α${\mathrm{-NiO}}_{x}$ obtained from AIMD simulations, where the shaded error bands represent the statistical uncertainty arising from averaging over multiple time origins. These plots show that, within the same system, the MSD slope increases with temperature, indicating that higher temperatures enhance the diffusion rate of Li^+^. Additionally, the temperature-dependent variation of the MSD slope differs among the investigated compositions, where α${\mathrm{-NiO}}_{1.33}$ exhibits the smallest fluctuation, while α-NiO and α${\mathrm{-NiO}}_{1.14}$ show comparable variations. It is also evident from the error bands that the uncertainty in the MSD becomes more pronounced at longer simulation times, which can be attributed to the reduced number of statistically independent samples available for averaging in the late diffusion stage. This behavior is consistent with the intrinsic statistical limitations of finite-time AIMD simulations. He et al. systematically studied the relationship between extracted diffusion characteristics and statistical variance in AIMD simulations, suggesting that the contributions from initial atomic vibrations should be excluded when evaluating diffusion coefficients [21]. Moreover, to further minimize statistical bias arising from both early-time non-diffusive motion and late-time sampling noise, the MSD within the time range of 0.1$t_{\max}$ to 0.7$t_{\max}$ is selected for the calculation of diffusion coefficients.

The diffusion coefficient was obtained from the slope of the mean square displacement (MSD) in the diffusive regime using the Einstein relation:

(2) $$D=\frac{1}{2d}\frac{dMSD(t)}{dt},$$

where d=3 for bulk systems.

The temperature dependence of the diffusion coefficient follows the Arrhenius relation:

(3) $$D=D_{0}e^{\left(-\frac{E_{b}}{kT}\right)},$$

allowing extraction of the activation energy $E_{b}$ from a linear fit of logD versus 1000/T. Based on this fitting, the diffusion coefficients at 300 K were extrapolated.

**Figure 3:**
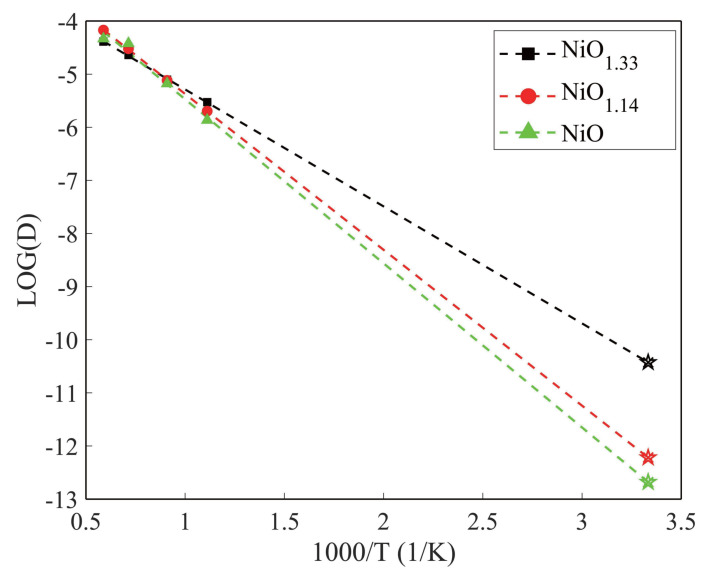
Arrhenius plot showing the temperature-dependent behavior of lithium-ion diffusivity ($D_{{\mathrm{Li}}^{+}}$) in α${\mathrm{-NiO}}_{x}$. The star symbols represent the diffusion coefficient at 300 K, which is obtained from the linear fit of ln($D_{{\mathrm{Li}}^{+}}$) versus 1000/T.

The diffusion barriers of Li^+^ in α${\mathrm{-NiO}}_{1.33}$, α${\mathrm{-NiO}}_{1.14}$, and α-NiO are 0.437 eV, 0.583 eV, and 0.614 eV, respectively, with extrapolated diffusion coefficients of $3.75\times 10^{-11}{\mathrm{cm}}^{2}$/s, $6.06\times 10^{-13}{\mathrm{cm}}^{2}$/s, and $2.09\times 10^{-13}{\mathrm{cm}}^{2}$/s at 300 K. It should be noted that Arrhenius extrapolation in amorphous systems has inherent limitations due to structural disorder and the presence of multiple local diffusion environments, which may lead to deviations from ideal Arrhenius behavior, particularly outside the simulated temperature range. In this work, the selected temperature range (900–1700 K) ensures sufficient sampling of Li^+^ diffusion events within the accessible AIMD timescale, and the MSD curves exhibit a clear linear regime, supporting the reliability of the extracted diffusion coefficients. Therefore, the obtained activation energies should be interpreted as effective values within this temperature range. The predicted diffusion coefficients at 300 K are in reasonable agreement with the experimentally measured value of $7.24\times 10^{-12}{\mathrm{cm}}^{2}$/s in NiO thin films [22], supporting the validity of the present approach. For comparison, $V_{6}O_{13}$ nanosheet films exhibit diffusion coefficients of ∼$10^{-12}$ ${\mathrm{cm}}^{2}$/s, which have been attributed to morphology-induced transport pathways [23]. In contrast, the present results demonstrate that amorphous ${\mathrm{NiO}}_{x}$ achieves comparable diffusion coefficients at room temperature, while the underlying mechanism is fundamentally governed by atomic-scale structural factors (Table 3 and Figure 3).

### 3.3. Local Structure of Amorphous α${\mathrm{-NiO}}_{x}$

**Figure 4:**
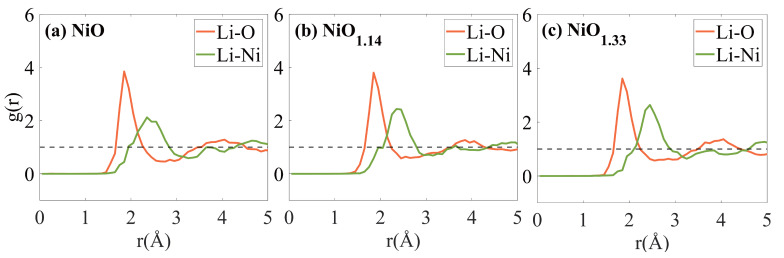
RDF of Ni-O in (**a**) α-NiO, (**b**) α${\mathrm{-NiO}}_{1.14}$, (**c**) α${\mathrm{-NiO}}_{1.33}$. The dashed line marks g(r) = 1, the average density.

The computational findings suggest that Li^+^ diffuses most rapidly in ${\mathrm{NiO}}_{1.33}$, followed by ${\mathrm{NiO}}_{1.14}$ and NiO, demonstrating a close correlation between the diffusion coefficient ($D_{{\mathrm{Li}}^{+}}$) and the material’s atomic structure. To better understand how the atomic structure affects the variation in Li^+^ diffusion properties, analyzing the local structural changes during diffusion is crucial. The radial distribution functions (RDF) for Li and Ni, O elements in α${\mathrm{-NiO}}_{x}$ at equilibrium are shown in Figure 4. Due to atomic interactions, the atomic density around bond lengths increases significantly. Therefore, in the RDF of α${\mathrm{-NiO}}_{x}$, the bond lengths between atoms can be identified at the first peak position. According to the findings in Figure 4a–c, the location of the initial peak can help establish the cutoff distance for Li-Ni and Li-O bonds. From the figure, it is evident that the cutoff distance for Li-O is 1.85 Å, while the cutoff distance for Li-Ni is 2.35 Å.

**Figure 5:**
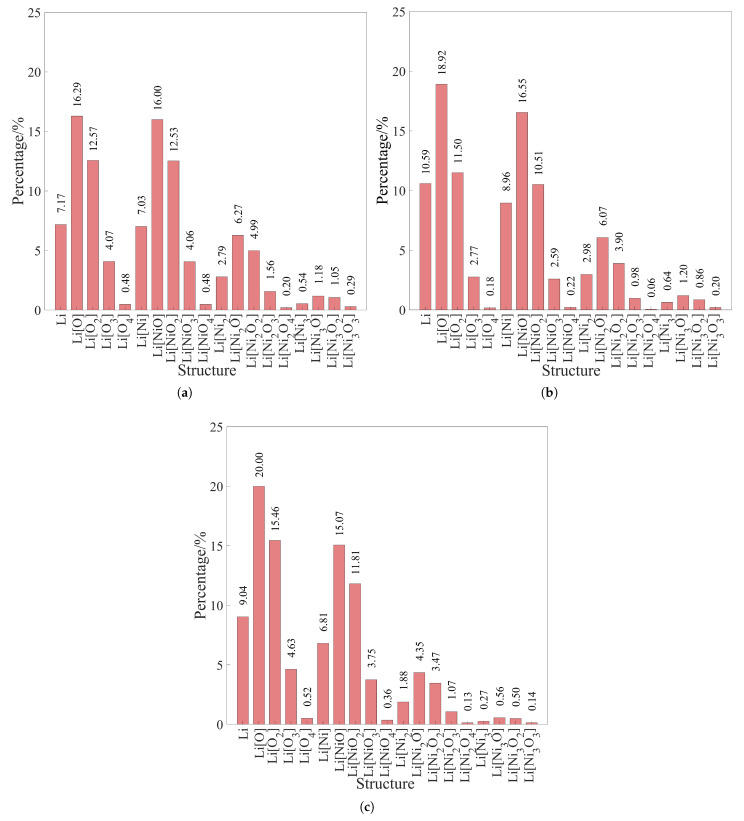
Statistics of local structures (Li[Ni_*x*_O_*y*_]) of (**a**) α-NiO, (**b**) α${\mathrm{-NiO}}_{1.14}$, (**c**) α${\mathrm{-NiO}}_{1.33}$.

The diffusion behavior of Li^+^ in amorphous ${\mathrm{NiO}}_{x}$ is strongly correlated with the evolution of its local coordination environment; however, these coordination motifs should be understood as manifestations of the underlying structural disorder rather than independent diffusion-controlling units. As shown in Figure 5a, the α-NiO structure is dominated by relatively well-defined Li-centered polyhedral environments such as Li[$O_{4}$], Li[${\mathrm{Ni}}_{3}$O], and Li[Ni_2_O_2_]. These relatively ordered coordination configurations reflect a more constrained local bonding network, which limits the availability of dynamically accessible migration pathways for Li^+^, resulting in lower diffusion coefficients. With increasing oxygen content, as illustrated in Figure 5b, the distribution of Li-centered coordination environments becomes more diverse, accompanied by a reduction in well-defined oxygen-coordinated polyhedra. This indicates an increasing degree of local structural disorder in the Ni–O framework. Such disorder leads to a broader distribution of local bonding strengths and coordination geometries, which effectively reduces the uniformity of the migration energy landscape. In α${\mathrm{-NiO}}_{1.33}$ (Figure 5c), the Li-centered environments become even more fragmented into smaller and less symmetric coordination motifs. This structural fragmentation enhances the connectivity between transient local configurations, thereby increasing the probability of Li^+^ accessing energetically favorable migration pathways. Importantly, these Li[Ni_*x*_O_*y*_] motifs should not be interpreted as isolated structural units directly controlling diffusion. Instead, the dominant factor governing Li^+^ transport is the degree of amorphous disorder in the Ni–O network. This disorder determines the distribution and connectivity of local coordination environments, and consequently shapes the effective migration energy landscape experienced by Li^+^. Overall, these results indicate that Li^+^ diffusion in amorphous ${\mathrm{NiO}}_{x}$ is primarily governed by a disorder-dominated mechanism rather than by specific local coordination motifs.

It should be noted that in amorphous systems, well-defined migration pathways and discrete site-to-site hopping mechanisms, as typically defined in crystalline materials, are not strictly applicable due to the absence of periodic symmetry and the continuous distribution of local environments. Therefore, Li^+^ transport in amorphous ${\mathrm{NiO}}_{x}$ is more appropriately described as a statistical hopping process arising from transient local configurations rather than migration along fixed atomic pathways. In this framework, Li^+^ diffusion is governed by a distribution of local hopping barriers associated with structural disorder in the Ni–O network.

## 4. Conclusions

This study employs first-principles calculations combined with SGCPMD simulations to construct amorphous ${\mathrm{NiO}}_{x}$ structures with varying oxygen content and systematically investigate the diffusion behavior of Li^+^. The results show that increasing oxygen content does not significantly alter the average Ni–O bond length, but leads to an increase in Ni coordination number and a higher degree of structural disorder in the Ni–O network. This structural evolution modifies the local bonding environment and affects the Li^+^ transport properties. The diffusion coefficients and migration barriers indicate that Li^+^ mobility is significantly enhanced with increasing oxygen content. Specifically, the diffusion barriers decrease from 0.614 eV in α-NiO to 0.583 eV in α${\mathrm{-NiO}}_{1.14}$ and further to 0.437 eV in α${\mathrm{-NiO}}_{1.33}$, accompanied by a corresponding increase in diffusion coefficients. Analysis of local coordination environments shows that increasing oxygen content leads to a broader distribution of Li-centered configurations and a reduction in well-defined polyhedral structures, reflecting enhanced amorphous disorder. Rather than being governed by specific Li[Ni_*x*_O_*y*_] motifs, Li^+^ diffusion is primarily controlled by the degree of structural disorder in the Ni–O network. This disorder determines the distribution and connectivity of local environments, giving rise to a heterogeneous energy landscape for Li^+^ migration. Therefore, Li^+^ transport in amorphous ${\mathrm{NiO}}_{x}$ is best described as a disorder-dominated diffusion process, in which transient local configurations collectively facilitate ion migration, rather than through well-defined diffusion pathways or specific structural units. These findings provide atomic-scale insight into the relationship between oxygen content, structural disorder, and Li^+^ transport, offering a atomic-scale understanding of Li^+^ transport processes that govern the switching kinetics of NiO-based electrochromic devices.

## Figures and Tables

**Table 1:** Comparison of density of α${\mathrm{-NiO}}_{x}$ with crystalline NiO.

Structure	a (Å)	b (Å)	c (Å)	Volume (${Å}^{3}$)	Density (${\mathrm{g/cm}}^{3}$)
α-NiO	13.080	13.080	8.720	1491.873	5.99
α ${\mathrm{-NiO}}_{1.14}$	12.780	12.780	8.526	1392.537	5.78
α ${\mathrm{-NiO}}_{1.33}$	12.538	12.538	8.359	1314.047	5.46
NiO	12.570	12.570	8.380	1324.081	6.74

**Table 2:** Comparison of average coordinate number and bond distances of Ni-O in α${\mathrm{-NiO}}_{x}$.

Structure	Ni-O Distance (Å)	Coordination Number	Method
α-NiO	1.875	3.792	SGCPMD
α ${\mathrm{-NiO}}_{1.14}$	1.875	4.362	SGCPMD
α ${\mathrm{-NiO}}_{1.33}$	1.875	4.716	SGCPMD
NiO	1.93	-	EXAFS [19]
${\mathrm{Li}}_{2}{\mathrm{NiO}}_{2}$	∼1.75	-	EXAFS [20]

**Table 3:** Comparison of Li^+^’s diffusion coefficients in α${\mathrm{-NiO}}_{x}$.

	Diffusivity (${\mathrm{cm}}^{2}$/s)		
Temperature (K)	α-NiO_1.33_	α-NiO_1.14_	α-NiO
900	$2.97\times 10^{-5}$	$2.02\times 10^{-5}$	$1.36\times 10^{-5}$
1100	$7.84\times 10^{-5}$	$7.63\times 10^{-5}$	$6.64\times 10^{-5}$
1400	$2.28\times 10^{-4}$	$3.00\times 10^{-4}$	$3.77\times 10^{-4}$
1700	$4.09\times 10^{-4}$	$6.74\times 10^{-4}$	$4.64\times 10^{-4}$

## Data Availability

The original contributions presented in this study are included in the article. Further inquiries can be directed to the corresponding author.
